# Qualitative Characteristics of Depression in Parkinson's Patients and Controls

**DOI:** 10.1155/2015/961372

**Published:** 2015-08-16

**Authors:** Cleo Kritzinger, Eva-Juliane Vollstedt, Katja Hückelheim, Anne Lorwin, Julia Graf, Sinem Tunc, Christine Klein, Meike Kasten

**Affiliations:** ^1^Department of Psychiatry and Psychotherapy, University of Lübeck, Ratzeburger Allee 160, 23538 Lübeck, Germany; ^2^Institute of Neurogenetics, University of Lübeck, Maria-Goeppert-Straße 1, 23562 Lübeck, Germany

## Abstract

*Background*. Depression is common in Parkinson's disease (PD); in light of typical PD pathology it may differ phenomenologically from depression in the general population. *Objective*. To assess depressive symptoms in PD patients and control groups and compare symptom profiles. *Methods*. After postal screening of 10,000 citizens of Lübeck, 642 participants were examined and the Beck Depression Inventory (BDI) was sufficiently answered by 477 subjects. Based on motor examinations, we distinguished PD patients, Healthy Controls (HC, no motor impairment), and Disease Controls (DC, motor impairment other than PD). *Results*. The sample comprised 331 men and 311 women, aged 65 ± 8 years. Out of the overall sample, 198 (41.5%) had a BDI score ≥9. BDI results above 9 points occurred in 34.5% of HC, 50.3% of DC, and 42.4% of PD patients. Compared to the control groups (HC, DC) the PD patients endorsed more “dissatisfaction” and “loss of appetite” but less “feelings of guilt,” “self-hate,” and “loss of libido.” *Conclusion*. Depressive symptoms are more frequent in PD patients compared to HC but not DC. Interestingly, the distribution of individual symptoms of the BDI differs between groups with an emphasis on loss of pleasure/enjoyment in the PD group, a symptom typically considered to be dopaminergically transmitted.

## 1. Introduction

Symptoms of depression are common in Parkinson's disease (PD) and are a major predictor of poor quality of life [1, 2]. However, prevalence and incidence rates of depression in PD vary widely, with 2.7–90% and 4–75%, partially due to differences in definition and assessment of depression [3]. The Diagnostic and Statistical Manual of Mental Disorders (DSM) [4] or International Classification of Disease (ICD) criteria offer clear definitions but introduce a relatively high severity threshold [5]. Clinical studies demonstrated negative effects of even subthreshold depression [6] on quality of life indicating their importance.

Studies on depression in PD usually apply rating scales; one of the recommended scales is the Beck Depression Inventory (BDI) [7]. The BDI covers several clinically important factors of depression, that is, mood, motivation, somatic symptoms, and negative cognition. Interestingly, previous studies indicate that depression in PD could be associated with fewer negative cognition and more somatic symptoms [6, 8]. However, these studies did not adjust for severity of depressive symptoms. This adjustment is important, as the qualitative characteristics of depression change with severity; for example, suicidal ideation is typical for severe depression.

In our study, we assessed depressive symptoms in PD patients and controls groups and compared the relative severities of the individual symptoms to provide symptom profiles.

## 2. Methods

Study participants were recruited via population-based screening. The aim of the screening was to recruit PD patients and control groups representative of the general population; the focus of the screening was on motor impairment. Between June 2010 and January 2011 we sent out screening questionnaires including 9 questions on symptoms of Parkinson's disease to 10,000 inhabitants of Lübeck, Germany, randomly selected by the residents' registration office after ethics approval of the local review board. Based on the 9-item screening questionnaire we assigned screening groups with no motor impairment (Healthy Controls) and motor impairment likely due to other reported disorders (Disease Controls), and we then invited a randomly selected and predefined number of participants for in-person examinations. All controls and 16 PD patients were recruited from the population screening and additional 91 PD patients were included from our outpatient clinic. This resulted in a total of 642 examinations. The detailed study design has previously been published [9] and includes annual follow-ups.

The in-person examination included neurological examinations using the Unified Parkinson Disease Rating Scale (UPDRS) [10] performed by experienced movement disorder specialists [11]. We applied several scales addressing nonmotor or psychiatric symptoms, among others the Mini Mental Status Examination (MMSE) and the Beck Depression Inventory (BDI). For the BDI, different cut-offs are being discussed; in order to achieve a high sensitivity we decided on a rather low cut-off of 9 points [12]. Based on the neurological examination and the UPDRS motor part, participants were grouped into “Healthy Controls” (HC, UPDRS III unremarkable), “Disease Controls” (DC, impairment in UPDRS III nonspecific for PD), and PD patients [11] according to the modified UK Brain Bank Criteria [13]. All subjects gave written informed consent before in-person examination (approved by the Ethics Committee of the University of Lübeck).

Statistical analyses were performed using SPSS 20. As histograms showed that data were not normally distributed, Mann-Whitney and Kruskal-Wallis tests were used to compare distribution of BDI items for symptom profiles in PD patients versus controls and men versus women. Chi-square tests were applied to compare frequencies, for example, of BDI severity groups. For symptom profiles we compared the frequency of items endorsed with at least 1 point (not corrected for multiple testing) for men versus women in all groups and, as separate analysis, for PD cases versus control groups. Two logistic regression models were built for the outcomes (1) BDI ≥ 9 and (2) suicidal ideation score ≥1 with age, sex, MMSE score, and group status (HC, DC, and PD patients) as possible predictors.

## 3. Results

The sample (*N* = 642) comprised 331 men and 311 women, aged 65 ± 8 years. Out of the overall sample, 477 answered the BDI sufficiently so it could be evaluated; of these 198 (41.5%) had a BDI score ≥9. BDI results above the 9-point cut-off occurred in 40.2% of men and 43.2% of women (*p* = 0.516, chi-square test). A BDI ≥ 9 points was present in 34.5% of HC, 50.3% of DC, and 42.4% of PD patients (*p* = 0.015, chi-square test) (Table 1).

**Figure 1:**
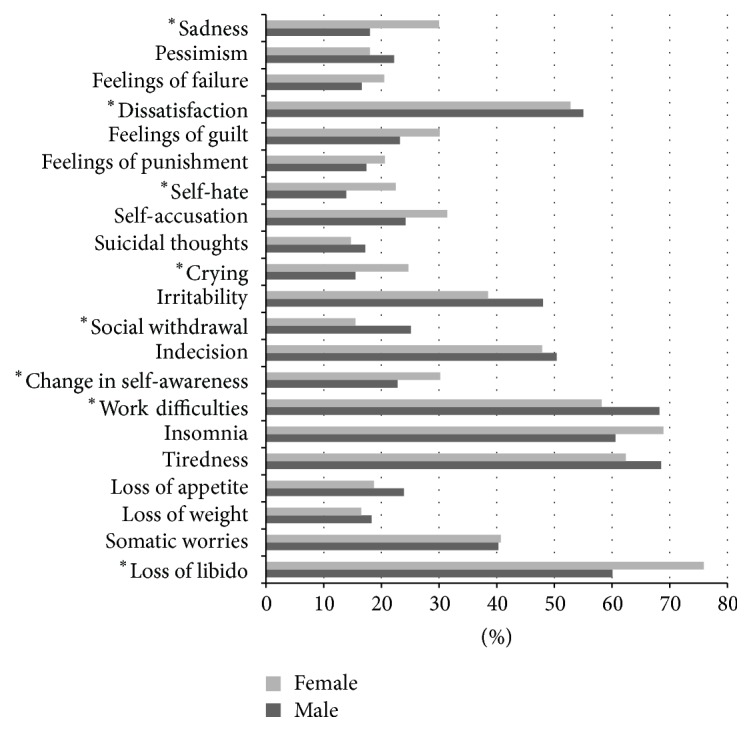
Symptom profile comparing male and female participants; percentage of subjects who answered the item with at least 1 point. ^*∗*^ *p* < 0.05, Mann-Whitney test.

Looking at the symptom profiles, differences between men and women included more frequent endorsement of the items “sadness,” “self-hate,” “crying,” “change in self-awareness,” and “loss of libido” in women. In contrast, men reported more “dissatisfaction,” “social withdrawal,” and “work difficulties” (all at *p* < 0.05, Mann-Whitney test, Figure 1).

**Figure 2:**
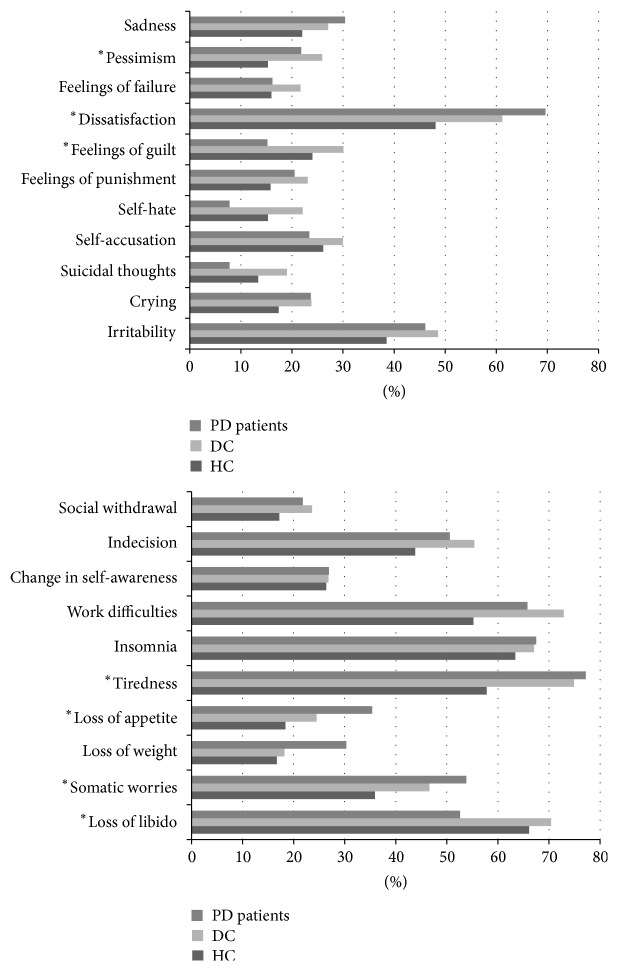
Symptom profile of PD patients, DC, and HC; percentage of subjects who answered the item with at least 1 point. ^*∗*^ *p* < 0.05, Kruskal-Wallis test.

Differences between the three groups “HC,” “DC,” and “PD patients” are depicted in Figure 2. In addition, we calculated differences between PD patients and combined control groups; here PD patients endorsed more “dissatisfaction” and “loss of appetite” but less “feelings of guilt,” “self-hate,” and “loss of libido” (all at *p* < 0.05, Mann-Whitney test).

In regression analysis for BDI ≥ 9, age and group assignment reached significance at *p* < 0.05 with younger age and assignment to DC group predicting higher BDI scores. In the model with suicidal ideation (at least thoughts of suicide without the intention to act) as outcome, older age was associated with more frequent endorsement of this item; DC reported suicidal ideation more frequently than the HC and PD patients, while sex and MMSE score as possible predictors did not reach significance (Table 2).

## 4. Discussion

An overall 37.5% of participants reported at least mild depressive symptoms, which clearly contrasts with population-based frequencies of depression between 16 and 18% [14]. We even had comparison data from a previous study performed in Lübeck, which applied DSM-IV criteria and reported a frequency of major depressive episodes of 10% [15].

In the context of this paper we used a BDI ≥ 9 as an indicator for depressive symptoms. This indicator is considered a low cut-off in clinical psychiatry but is recommended for screening for depression in PD, offering a high sensitivity which was intended in our study. Consequently, subjects with only mild symptoms who may not fulfill clinical criteria of depression were included [12]. As expected, PD patients reported depressive symptoms more frequently than HC (42% to 35%). Interestingly, if controls with other disorders causing some motor impairment are considered, the figures change to 42% in PD patients and 50% in DC.

This result is intriguing as depression is vividly discussed as a possible early marker for PD [16] and depression as an integral part of PD would be biologically plausible [2]. In the field of PD research, major efforts address the earlier diagnosis.

Currently PD is diagnosed via its cardinal motor signs [17], that is, rigidity, bradykinesia, rest tremor, and postural instability. However, upon manifestation of these signs more than 50% of the dopaminergic neurons are lost [18]. This is a major limitation for any approach of neuroprotection. In addition to the loss of dopaminergic innervation in PD, the serotonergic and noradrenergic systems are affected [2]. This is one line of argument for depression as a possible consequence of PD pathology. However, our data suggest a more general association between motor impairment and depressive symptoms as we also observed an increased likelihood of depression in participants of the DC group. In our study, the HC group did not have motor impairment but could have diseases such as hypertension or diabetes, whereas the DC had some motor impairment that was not typical of PD (e.g., slow movements but not decreasing amplitude) and other disorders that could explain the impairment (e.g., arthritis). This link between motor impairment and depression needs to be confirmed and followed in additional studies. In logistic regression, we observed impact of “other diseases” on likelihood of BDI ≥ 9; this clearly indicates the importance of using controls representative of the general population and including other disorders.

Another aspect apart from frequency is the clinical picture of depression. Here, the predominance of dopaminergic affection in PD pathology raises the question if depression in PD shows distinct features. The BDI item inquiring if people are able to enjoy themselves is item 4, often abbreviated as “dissatisfaction.” This item seems most closely related to feelings of pleasure and reward that may be interpreted as dopaminergically transmitted. Interestingly, this item was more frequently endorsed by PD patients than both control groups (*p* = 0.015). The other item with higher frequency in PD is “loss of appetite” (*p* = 0.023), an item typically associated with PD even in the absence of depression. In a previous study named “Genepark” we had observed the same effect with “loss of enjoyment” being a prominent feature [19].

## 5. Conclusions

(1) The role of motor impairment as a contributing factor for the development of depressive symptoms in subjects with and without PD needs further evaluation. From the perspective of mere frequencies, the association between PD and depression seems less clear if other diseases are considered. (2) Description of depression characteristics based on the BDI indicates that dopaminergically transmitted symptoms, namely, loss of pleasure/enjoyment, may be predominantly affected in PD.

## Figures and Tables

**Table 1:** Group characteristics—clinical and demographic.

	HC	DC	PD patients	*p* value
	*n* = 282	*n* = 253	*n* = 107	Chi-square/Kruskal-Wallis
Male	141 (50%)	122 (48.2%)	68 (63.6%)	0.009
Female	141 (50%)	131 (51.8%)	39 (36.4%)	
Age	64 ± 7 (95% CI 62.77–64.43)	67 ± 7 (95% CI 65.96–67.72)	67 ± 10 (95% CI 64.89–68.75)	<0.001^*∗*^
BDI available	232 (82%)	179 (71%)	66 (62%)	
BDI < 9	152	89	38	0.016
%°	65.5	49.7	57.6	
95% CI	59.4–71.6%	42.4–57.0%	45.7–69.5%	
BDI 9–12	32	34	7	
%°	13.8	19.0	10.6	
95% CI	9.4–18.2%	13.3–24.8%	3.1–18.0%	
BDI 13–17	23	23	13	
%°	9.9	12.9	19.7	
95% CI	6.1–13.7%	8.0–17.8%	10.1–29.3%	
BDI ≥ 18	25	33	8	
%°	10.8	18.4	2.1	
95% CI	6.8–14.8%	2.7–24.0%	4.2–20.0%	
MMSE	28 ± 2 (95% CI 28.07–28.43)	27 ± 2 (95% CI 27.09–27.65)	27 ± 2 (95% CI 26.73–27.63)	<0.001^*∗*^

^*∗*^Kruskal-Wallis test for continuous data.

°Percentage of BDI data available over groups.

CI: confidence interval.

**Table 2:** Overview of possible predictors for a BDI outcome ≥9 and for a suicidal ideation score ≥1.

Predictor	OR	95% CI
(1) Logistic regression model: outcome BDI ≥9		
Age (older)	0.957	0.93–0.98
Group (compared to HC)		
DC	2.107	1.39–3.21

(2) Logistic regression model: suicidal ideation score ≥1		
Age (older)	0.955	0.93–0.99
Group (compared to HC)		
DC	1.776	1.06–2.97
